# TCF3::HLF orchestrates an enhancer-promoter network with activation of MEF2C to promote immature HSC gene expression in leukemia

**DOI:** 10.1126/sciadv.adu3728

**Published:** 2026-05-06

**Authors:** Valdemar Priebe, Bartimée Galvan, Aneta Drakul, Nicola Margelisch, Júlia Aguadé-Gorgorió, Kaivalya Walavalkar, Yun Huang, Hanna K. A. Mikkola, Beat Bornhauser, Raffaella Santoro, Jean-Pierre Bourquin

**Affiliations:** ^1^ Division of Oncology and Children’s Research Centre, University Children’s Hospital Zurich, Zurich, Switzerland.; ^2^ Department of Molecular Mechanism of Disease, University of Zurich, Zurich, Switzerland.; ^3^ Department of Molecular, Cell and Developmental Biology, University of California, Los Angeles, Los Angeles, CA, USA.; ^4^ Eli and Edythe Broad Center for Regenerative Medicine and Stem Cell Research, University of California, Los Angeles, Los Angeles, CA, USA.; ^5^ Jonsson Comprehensive Cancer Center, University of California, Los Angeles, Los Angeles, CA, USA.; ^6^ Molecular Biology Institute, University of California, Los Angeles, Los Angeles, CA, USA.

## Abstract

Oncogenic fusion transcription factors (TFs) frequently drive hematopoietic malignancies by altering gene expression in key developmental programs. TCF3::HLF is a fusion TF that characterizes a rare, treatment-resistant subtype of B cell acute lymphoblastic leukemia [t(17;19) TCF3::HLF-positive B-ALL]. Despite its clinical significance, the mechanisms by which TCF3::HLF induces leukemia are unclear. We used HiChIP mapping and genetic interference to analyze TCF3::HLF at the 3D genome level, revealing enhancer-promoter interactions that control gene activation or repression. Notably, TCF3::HLF directly regulates *MEF2C* expression through its enhancer, as interference disrupted *MEF2C* transcription and inhibited leukemia propagation. This disruption also diminished embryonal hematopoietic stem cell (HSC) gene signatures and restored mature HSC and B-lymphoid markers. These findings highlight *MEF2C* as a critical component of the transcriptional network reprogrammed by TCF3::HLF. Our study provides insight into how TCF3::HLF rewires the 3D genome to drive leukemia and serves as a resource for further exploration of the TCF3::HLF regulome.

## Teaser

TCF3::HLF fusion protein mediates 3D genomic interactions in t(17;19)-positive acute lymphoblastic leukemia

## INTRODUCTION

Studies of the three-dimensional (3D) genome have provided relevant insights into gene regulation that cannot be described in direct linear fashion, such as enhancer-promoter (E-P) contacts (*1*–*3*). Increasing evidence shows that enhancer malfunction is a key process that drives the aberrant regulation of oncogenes in cancer. Differential genome organization between healthy and malignant cells has been observed and can among other be attributed to chromosomal translocations leading to both alterations of genome structure or hijacking of enhancers by oncogenic fusion proteins (*4*, *5*).

Chromosomal translocations resulting in oncogenic fusion of transcription factors (TFs) involving hematopoietic master regulators are frequent genetic abnormalities in acute leukemias (*6*–*8*). These chimeric TFs may take over the functions of master regulators that typically control gene expression programs defining cellular identity by hijacking enhancer elements in cooperation with other TFs (*8*–*10*). These oncogenic TFs disrupt fundamental cellular processes, including self-renewal, differentiation, and proliferation, thereby imparting leukemogenic potential to hematopoietic stem and progenitor cells (HSPCs) (*11*).

The chromosomal translocation t(17;19)(q22;p13) generates the fusion protein TCF3::HLF that defines a rare subtype of B cell acute lymphoblastic leukemia [t(17;19) TCF3::HLF-positive ALL] (*12*–*15*). This chimera consists of the transactivation domains of the transcription factor 3 (TCF3 or E2A), a TF that drives lymphoid development, fused to the DNA binding and dimerization domains of the leukemia-associated TF hepatic leukemia factor (HLF), which is a regulator of multipotent hematopoietic progenitors and is rapidly down-regulated upon differentiation (*14*, *16*–*19*). Accordingly, the resulting chimeric TCF3::HLF TF rewires the transcriptional programs toward a stem-like/myeloid expression lineage profile and promotes leukemogenesis in cells of committed lymphoid origin (*20*, *21*). Recent data have shown that TCF3::HLF fusion is essential for disease maintenance, as depletion of TCF3::HLF resulted in marked reductions in DNA synthesis and cell viability, which, however, did not associate with a prominent increase of apoptosis markers (*20*). Although inclusion of the BCL2 inhibitor venetoclax and of CD19-directed immunotherapy resulted in sustained remissions (*21*, *22*), TCF3::HLF-positive ALL still remains one of the most resistant ALL subtypes.

Previous chromatin immunoprecipitation (ChIPseq) analyses of TCF3::HLF in t(17;19)-positive ALL cells showed preferential association of TCF3::HLF with enhancer sequences (*20*). However, knowledge about the direct transcriptional targets of TCF3::HLF remains limited, given the difficulty to predict distant interactions between regulatory elements in the genome. To capture the relevant clusters of direct TCF3::HLF target genes, we performed TCF3::HLF-HiChIP to identify the 3D architecture of TCF3::HLF bound E-P interactions that is underlying the oncogenic activity in t(17;19)-positive ALL. By integrating TCF3::HLF spatial genomic information with chromatin and transcriptomic data, we identified networks of activating and repressing E-P interactions that are directly driven by TCF3::HLF. This approach identified previously undetected TCF3::HLF target genes that are specifically up-regulated in t(17;19)-positive ALL relative to other ALL types and implicated in cell-cell interaction or neuronal development. The most prominent functional TCF3::HLF-bound E-P interaction was with the myocyte enhancer factor 2C (*MEF2C*). We demonstrated that *MEF2C* depends on the interaction of TCF3::HLF with the enhancer and showed that it controls substantial portions of the TCF3::HLF transcriptional program, mirroring fetal hematopoietic stem cell (HSC) features with functional essentiality for the leukemia. These findings represent a paradigm for how oncogenic fusion TFs can rewire the 3D genome to drive malignancy.

## RESULTS

### A map of TCF3::HLF 3D genomic interactions in t(17;19)-positive ALL cells

**Fig. 1.**
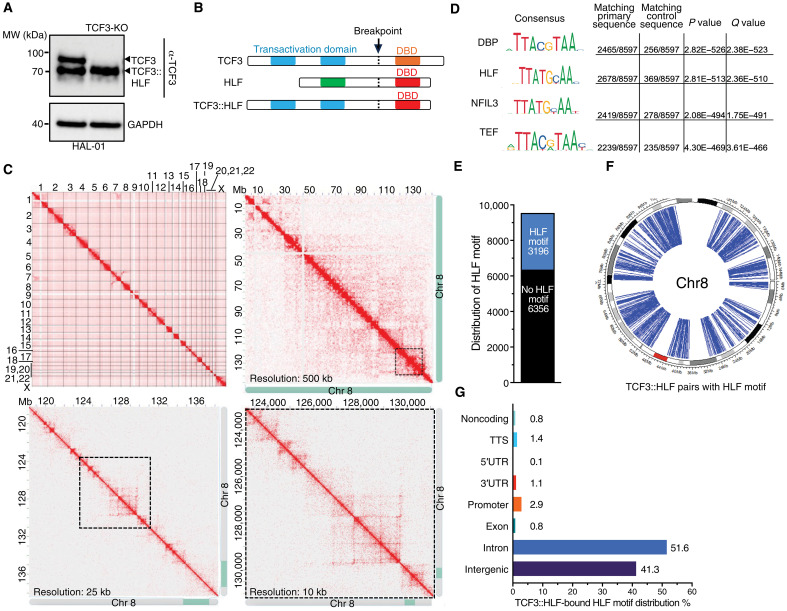
TCF3::HLF 3D genomic interactions in t(17;19)-positive ALL cells. (**A**) Anti-TCF3 immunoblot of HAL-01 cells expressing TCF3::HLF. Endogenous TCF3 was knocked out by CRISPR editing using sgRNA. Expression of TCF3 and TCF3::HLF are shown, glyceraldehyde-3-phosphate dehydrogenase (GAPDH) serves as loading control. (**B**) Schematic of *TCF3*, *HLF*, and *TCF3::HLF* translocation products. DBD: DNA binding domain. (**C**) HiChIP of TCF3::HLF showing genome contacts of all chromosomes and of chromosome 8 at different resolutions. Data were visualized with Juicebox (*80*). (**D**) Motif enrichment of HiChIP-detected TCF3::HLF binding sites (*n* = 9552). (**E**) Distribution of HiChIP-detected TCF3::HLF binding sites containing HLF motif. (**F**) Circular ideogram of chromosome 8 showing the distribution of TCF3::HLF loops (blue arcs) with at least one HLF motif. (**G**) Genomic annotation of HiChIP-detected TCF3::HLF binding sites containing HLF motif. 5′UTR, 5′ untranslated region; 3′UTR, 3′ untranslated region.

To map the 3D genomic interactions associated with the fusion protein TCF3::HLF in t(17;19) ALL cells, we performed HiChIP, a method that combines ChIP and chromosome conformation capture (HiC) to directly capture long-range DNA interactions associated with a protein of interest (*23*). To perform the HiChIP, we used the TCF3::HLF-positive cell line HAL-01 (*12*, *20*), whose TCF3 wild-type allele was stably knocked out, thereby enabling selective immunoprecipitation of the fusion protein when using antibodies against TCF3 (Fig. 1A). The loss of TCF3 in HAL-01 cells should not affect the genomic association of TCF3::HLF as the DNA binding domain (DBD) of the fusion protein is confined to the HLF region and lacks the TCF3-DBD (Fig. 1B). Moreover, TCF3–knockout (KO) HAL-01 cells and the parental HAL-01 cell line showed similar TCF3::HLF genomic occupancy (fig. S1, A and B), gene expression profiles (fig. S1C), and cell proliferation (fig. S1D). All these results indicate that the TCF3-KO HAL-01 cell line constitutes a suitable model for the HiChIP analysis to study TCF3::HLF-positive B-ALL.

We generated TCF3::HLF-HiChIP maps from two independent replicates that showed high concordance and identified >49,000 significant interaction pairs [false discovery rate (FDR) < 0.01] distributed across the 23 human chromosome pairs (fig. S1E and tables S1 and S2). Interactions have been identified with the loop calling tool FitHiChIP (for details, see Materials and Methods) (*24*). These interaction pairs comprised 9552 primary peaks that exhibit a significant enrichment in the DNA recognition motif of basic leucine zipper (*bZIP*) TFs, including *HLF* motif that is recognized by TCF3::HLF (*P* = 2.81 × 10^−513^) (Fig. 1D). Among these primary peaks, 3196 (33%) contained an HLF binding motif, underscoring the specificity of TCF3::HLF-HiChIP (Fig. 1, E and F). The rest of the sites lacking HLF motifs showed a significant enrichment in the DNA recognition motif of several zing-finger proteins, suggesting a potential interconnection between TCF3::HLF and other TFs (table S3). However, given that TCF3::HLF binds to HLF motifs, we restricted our analysis to highly confident interaction pairs in which TCF3::HLF binding sites contain HLF motif. The TCF3::HLF-bound regions with HLF motif were predominantly located at intergenic regions (42%) and introns (52%), with only a small fraction (2.9%) falling within gene promoter regions (Fig. 1G), a result consistent with previous TCF3::HLF-ChIPseq data (*20*).

**Fig. 2.**
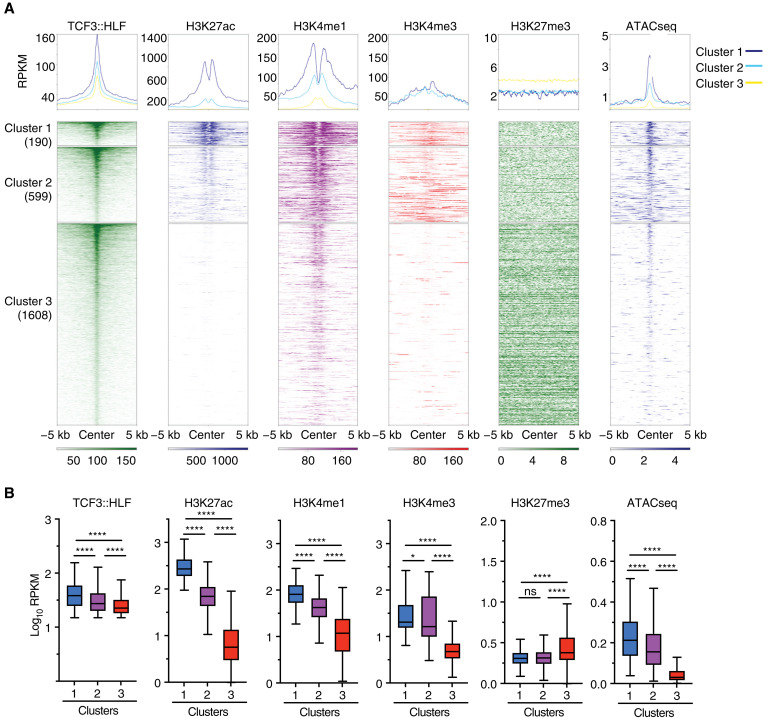
Chromatin features of TCF3::HLF 3D genomic interactions with HLF motif. (**A**) Heatmaps of TCF3::HLF, H3K27ac, H3K4me1, H3K4me3, and H3K27me3 occupancy and chromatin accessibility by ATACseq (*74*) at HLF motif of parental HAL-01 cells. Sequences were clustered in three groups based on the histone modification content. (**B**) Boxplots showing the average RPKM read density of regions centered on HLF motifs. Statistical significance (*Q* values) was calculated with a two-tailed *t* test, followed by Benjamini-Hochberg correction (*P < 0.05 and ****P < 0.0001; ns, not significant).

Next, we analyzed the chromatin state of these regions containing the HLF binding motif by assessing the occupancy of active (H3K27ac, H3K4me3, and H3K4me1) and repressive (H3K27me3) histone modifications and chromatin accessibility [Assay for Transposase-Accessible Chromatin using sequencing (ATACseq)] in the parental leukemia cell line HAL-01 (Fig. 2A). We first selected the top 75% TCF3::HLF-enriched regions at the HLF motif. As expected, we observed high signal for active histone modifications and chromatin accessibility at TCF3::HLF-bound sites, whereas there was no evident enrichment for H3K27me3, indicating that these sequences display active chromatin states. The presence of sharp peak signals for H3K27ac and H3K4me1 relative to H3K4me3 at HLF motif recalls an enhancer chromatin signature. Hierarchical clustering defined three groups according to the levels of the analyzed histone modifications (Fig. 2, A and B). Cluster 1 displayed higher enrichment for H3K27ac and H3K4me1 and higher chromatin accessibility than clusters 2 and 3. Cluster 2 was enriched in H3K4me1 while showing lower H3K27ac content relative to cluster 1, a chromatin signature characterizing primed or poised enhancers (*25*). Consistent with this, we observed a modest but significant negative correlation (*r* = −0.22; *P* = 1 × 10^*−*8^) between chromatin accessibility (ATACseq) and TCF3::HLF occupancy (ChIPseq) at these sites, whereas cluster 1 showed no significant correlations, likely due to its strong enrichment in active chromatin modifications (fig. S1F). Cluster 3 was mostly depleted of active histone modifications and enriched for H3K27me3 yet displayed higher chromatin accessibility together with elevated TCF3::HLF occupancy, suggesting that these sequences could be embedded in a repressive chromatin state that can become more accessible by high level of TCF3::HLF occupancy.

**Fig. 3.**
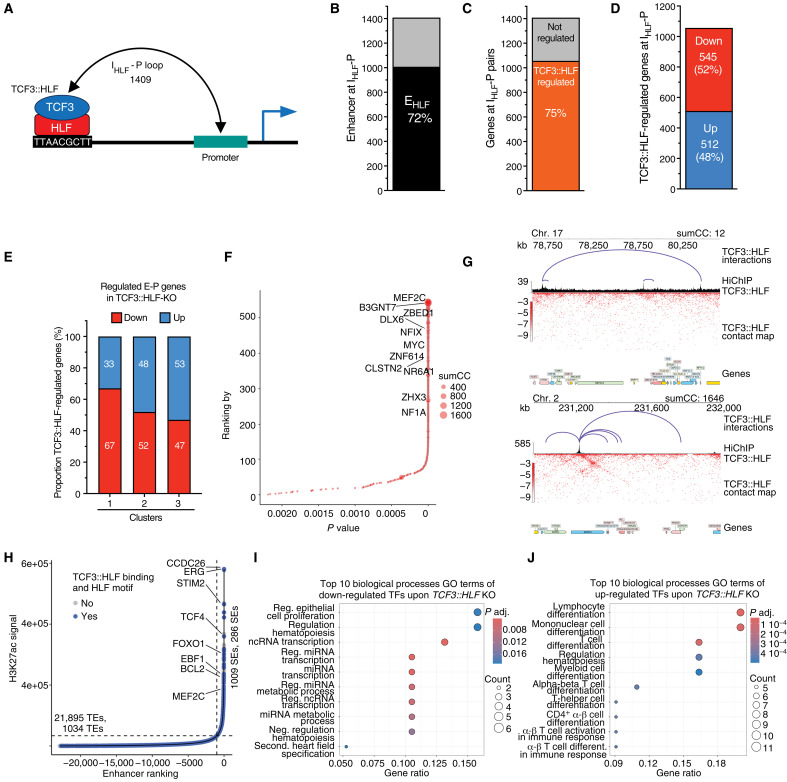
Enhancer-promoter pairs mapped with HiChIP identify TCF3::HLF direct target genes. (**A**) Illustration of I_HLF_-P interaction pairs that are bound by TCF3::HLF and contain HLF motif. (**B**) Number of I_HLF_-P interaction containing enhancer sequences (E_HLF_). (**C**) Number of TCF3::HLF regulated genes at I_HLF_-P pairs (E-P genes). (**D**) Number of significantly up-regulated and down-regulated E-P genes upon TCF3::HLF-KO. (**E**) Distribution of up-regulated and down-regulated E-P genes among the three clusters obtained with the histone modification analysis shown in Fig. 1F. (**F**) Plot showing the P value and the ranked order of interaction of I_HLF_-P pairs for the 1057 E-P genes significantly regulated upon TCF3::HLF-KO. The size of the data points indicates the cumulative interaction value (sumCC). (**G**) Representative contact maps with low and high sumCC values. (**H**) Plot showing the ranked order of H3K27ac signal using ROSE analysis. Typical enhancers (TEs) and super enhancers (SEs) are annotated as sites with TCF3::HLF binding (blue) or not (gray). Enhancers regulating an E-P gene of interest according to HiChIP are annotated with target gene. (**I** and **J**) Plots showing Gene Ontology terms for biological processes of TFs from E-P genes that are down-regulated (I) and up-regulated (J) upon TCF3::HLF-KO.

To identify genes directly regulated by TCF3::HLF, we focused on TCF3::HLF-bound inter- or intragenic regions containing the HLF motif (I_HLF_) interacting with promoters (P) (*n* = 1409) (Fig. 3A). Using Rank Ordering of Super-Enhancers (ROSE) (*26*, *27*) and the Fantom5 database (*28*, *29*), we found that a large majority of these I_HLF_-P interactions (72%) correspond to enhancers, a result consistent with the analysis of the histone modifications (Fig. 2 and table S1). Only 1% of these pairs contains an HLF motif at both anchors, indicating that heterotypic interactions are strongly predominant. By integrating previously published RNA sequencing (RNA-seq) data of the parental leukemia cell line HAL-01 upon *TCF3::HLF*-KO (*20*), we found 1057 (75%) I_HLF_-P pairs corresponding to the promoters of genes significantly up-regulated [512, adjusted *P* (adj. *P*) < 0.05] or down-regulated (545, adj. *P* < 0.05) upon TCF3::HLF loss (from now on named E-P genes; Fig. 3, C and D, and table S4). Among the E-P genes, only 32 involved contacts with HLF motifs at both anchors, a result consistent with previous ChIPseq results showing that only a small fraction of TCF3::HLF associates with promoter regions (*20*). Of these genes, approximately half were up-regulated and half down-regulated upon TCF3::HLF-KO, showing no consistent association with gene expression changes. Next, we analyzed the distribution of up- and down-regulated E-P genes in the three clusters obtained from the analysis of histone modifications at I_HLF_ regions bound by TCF3::HLF (Fig. 3E). We found that 67% of E-P genes within cluster 1 were down-regulated upon TCF3::HLF loss, indicating an association between TCF3::HLF and the expression of these genes, potentially mediated through its interaction with active enhancers and 3D contacts with the corresponding promoters. In contrast, we did not find differences in the distribution of up- and down-regulated genes in clusters 2 and 3.

To identify E-P genes with the most significant I_HLF_-P interaction, we first calculated the sum of contact counts (sumCC), a value that represents the cumulative counts of all significant I_HLF_-P interactions and consequentially their contact frequency (Fig. 3, F and G, and table S1). By using this approach, we identified not only the most significant I_HLF_-P interactions but also the ones with highest contact frequency. Among these I_HLF_-P interactions, the most significant was between the promoter of *MEF2C* and the TCF3::HLF-bound sequence that was recognized as enhancer by the ROSE and FANTOM dataset analysis. MEF2C is a regulator of hematopoietic self-renewal and differentiation, and it has been associated with increased risk of relapse when highly expressed in several subtypes of AML (*30*–*35*). However, its role in TCF3::HLF-positive ALL has not yet been investigated, a task that we set to investigate in this study (see below). Consistent with previous work (*20*), we also detected a *MYC* E-P interaction as one of the highest interactions within the E-P genes. Furthermore, among the E-P genes positively regulated by TCF3::HLF, we found *BCL2* (Fig. 3H and tables S4 and S5). This result is consistent with recurrent high sensitivity observed with the BCL2-specific BH3-mimetic venetoclax in the clinic, which is now recommended for use in combination chemotherapy in first line for TCF3::HLF-positive leukemia (AIOEP-BFM 2017 study, EudraCT 2016-001935-12) (*20*, *21*).

To obtain insight into the possible network of transcriptional regulators that are directly controlled by the TCF3::HLF, we identified 94 TFs among E-P genes that were significantly regulated upon *TCF3::HLF*-KO (39 down-regulated and 55 up-regulated; table S6). Among the 39 TFs that are down-regulated by TCF3::HLF-KO and detected in active clusters 1 and 2 based on chromatin marks, we found MEF2C, MYC, MESP1, and DLX6 genes among others, which regulate proliferation, miRNA metabolism, and contextual repression of hematopoiesis (Fig. 3I and table S6) (*31*, *34*, *36*–*40*). Among the 55 TF genes that are up-regulated by TCF3::HLF-KO, we found HLX, SOX4, FOXP1, and BCL6, which regulate hematopoiesis and myeloid differentiation (*41*–*46*) (Fig. 3J and table S6). These results suggest that TCF3::HLF might regulate a network of TFs to modulate the cellular state in critical functional hallmarks of cancer such as cell proliferation and self-renewal.

We next explored which of the direct targets that are activated by TCF3::HLF would be specifically expressed in TCF3::HLF ALL compared to all other ALL subtypes. We took advantage of a comprehensive dataset containing >10,000 pediatric patients with cancer and long-term survivors (www.stjude.cloud) (*47*) to compare the expression of E-P genes among 24 ALL subtypes. We identified 22 E-P genes that are preferentially expressed in TCF3::HLF ALL, providing further candidate biomarkers for this leukemia subtype (fig. S2A). Gene Ontology analysis for biological processes shows involvement of cell-cell adhesion (fig. S2B and table S7). Among the most specifically expressed genes, we detected CLSTN2 and B3GNT7 (Fig. 3F, fig. S3, A to D, and table S7). Both genes encode for membrane proteins with possible roles for cell adhesion, invasion, and metastasis, with a role in neuronal development (*48*, *49*). These warrant further functional investigation for possible mechanisms of action and targets for intervention. Together, 3D conformational mapping provides new insights into candidate direct targets of TCF3::HLF and the 3D organization of the TCF3::HLF oncogenic program.

### TCF3::HLF builds a hub of long-range interactions between super-enhancer elements and *MYC* promoter to drive MYC expression

**Fig. 4.**
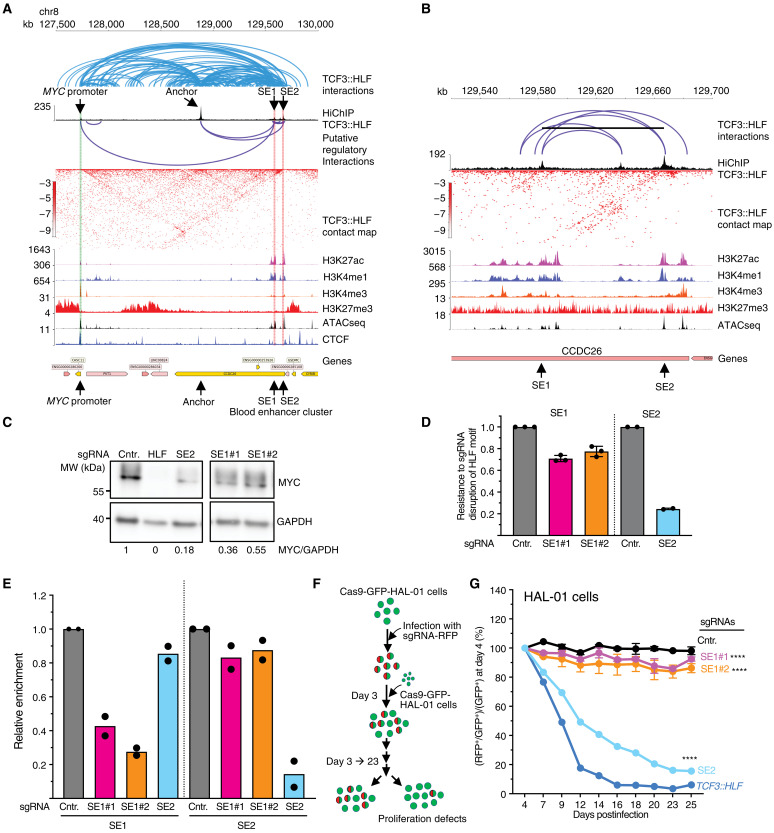
3D genome interactions bound by TCF3::HLF at MYC promoter and BENC locus. (**A**) Visualization of TCF3::HLF genomic interactions at the MYC and BENC locus and the corresponding TCF3::HLF-HiChIP, H3K27ac, H3K4me1, H3K4me3, and H3K27me3 ChIPseq and ATACseq profiles. TCF3::HLF interactions identified by HiChIP are depicted as arcs. Interactions among regions with HLF motif are depicted with viola arcs. (**B**) Magnification of TCF3::HLF-HiChIP profile at the BENC locus. TCF3::HLF interactions are depicted as arcs. (**C**) Western blot showing MYC expression in HAL-01 cells after 4 days infection with sgRNA-Control and sgRNAs targeting TCF3::HLF (HLF) or the HLF motif at SE2 or SE1 sequences. GAPDH serves as loading control. Values represent MYC levels relative to GAPDH and were normalized to the control sample. (**D**) sgRNA efficiency for HLF motif disruption was measured by quantitative polymerase chain reaction (qPCR) using primers encompassing SE1 or SE2 sequences, followed by normalization relative to the nontargeting control (sgCntr.). Values are three independent experiments. Data are presented as means ± SD. (**E**) ChIP-qPCR for TCF3::HLF was performed using TCF3 antibodies and Cas9-expressing HAL-01 cells with sgRNAs targeting SE1, SE2, and sgRNA-Control. Values were normalized to input and TCF3:HLF enrichment in cells treated with sgRNA-Control (Cntr.). Data are from two independent experiments. (**F**) Experimental scheme of the competitive assay. HAL-01 cells expressing Cas9-GFP were transduced with RFP-coexpressing sgRNA vectors targeting SE1, SE2, or *TCF3::HLF* and and sgRNA-Control. (**G**) Competitive assay quantification in HAL-01 cells upon disruption of SE1 (sgRNA-SE1#1 and #2), SE2, and TCF3::HLF with the corresponding sgRNAs. Values correspond to the ratio between RFP^+^/GFP^+^ (with sgRNA) and GFP^+^ (no sgRNA) cell number at the indicated days relative to the ratio between RFP^+^/GFP^+^ and GFP^+^ cell number measured at day 4 posttransduction. Statistical significance (*P* values) of two independent experiments was calculated with two-way repeated measures analysis of variance (ANOVA) (****P < 0.0001).

To further assess the accuracy of the TCF3::HLF-HiChIP, we examined the *MYC* locus, a well-established example of TCF3::HLF-mediated regulation within the 3D genome (*20*). We have previously reported that TCF3::HLF associates with the super enhancer 2 (SE2) within the blood enhancer cluster (BENC) to regulate *MYC*, as disruption of the HLF motif in SE2 reduced its interaction with the *MYC* promoter and down-regulated *MYC* expression (*20*). Notably, the disruption of SE2 mimics the effects of *MYC* KO, impairing leukemia maintenance and propagation. HiChIP data not only validated this interaction but also provided additional TCF3::HLF chromatin anchors and binding sites (Fig. 4A). First, we observed TCF3::HLF HiChiP signal at *MYC* promoter that was not detectable in previous TCF3::HLF-ChIPseq analyses, supporting a direct role of TCF3::HLF in directly regulating *MYC* expression. Second, we identified additional TCF3::HLF binding sites upstream of and within the Blood Enhancer Cluster (BENC) locus, thereby constituting a network of interactions involving the *MYC* promoter. One strong TCF3::HLF position was detected upstream and outside of the annotated BENC enhancer sequences (Anchor, Fig. 4A). This site displays interactions with both TCF3::HLF binding sites at the BENC and *MYC* promoter. In addition, this anchor region is close to a CTCF binding site and depleted of H3K27ac while enriched in H3K4me1, which is proposed to mark enhancer poised states, and is categorized within cluster 3 in our histone state analysis (Figs. 2 and 4A) (*50*, *51*). This anchor site might be involved in stabilizing the 3D structure of this regulatory domain, although this remains to be determined. In the absence of a suitable PAM sequence, we could not target it using CRISPR-Cas9 system, and PAM-independent detection system will be required to address the function of this type of HLF binding motifs in the genome.

Notably, we also found an additional binding site within the BENC locus, SE1, which interacts with SE2 and *MYC* promoter (Fig. 4, A and B). Both SE1 and SE2 were enriched for H3K27ac and H3K4me1, the chromatin signature of active enhancers. To determine whether SE1 can also be implicated in *MYC* regulation, we disrupted the HLF site of SE1 in HAL-01 cells by infection with CRISPR constructs expressing single guide RNA (sgRNA) targeting this sequence. As controls, we used sgRNA targeting SE2 and TCF3::HLF that were previously shown to reduce MYC transcription and an sgRNA not targeting the human genome (sgRNA-Control) (*20*). After 4-day infection, we measured MYC expression by Western blot. Consistent with previous results (*20*), targeting SE2 and TCF3::HLF sequences reduced MYC expression. The disruption of SE1 also led to decreased MYC levels, although the effect was less pronounced than that observed upon SE2 and TCF3::HLF disruption (Fig. 4C). This difference is likely due to a lower sgRNA efficiency in targeting and disrupting the HLF motif within SE1 compared to SE2 (Fig. 4D). Accordingly, the disruption of the HLF motif at SE2 led to an almost sevenfold reduction in TCF3 binding, while the disruption at SE1 also reduced TCF3 association, although less strongly, by approximately two- to fourfold (Fig. 4E). To determine whether SE1 can have an impact on cell proliferation as previously shown for SE2 (*20*), we performed a cell competitive assay in HAL-01 cells that were engineered for stable Cas9–green fluorescent protein (GFP) expression. Cells were transduced with a lentiviral vector expressing red fluorescent protein (RFP) and sgRNAs targeting SE1, SE2, or *TCF3::HLF* and an sgRNA-Control (Fig. 4, F and G). Cell proliferation was quantified by flow cytometry by measuring the number of GFP^+^ and RFP^+^ cells from day 4 after infection until day 23. Consistent with previous findings (*20*), the disruption of either SE2 or *TCF3::HLF* markedly impaired cell proliferation. Targeting SE1 resulted in only a mild, although statistically significant, reduction in growth. This modest effect may reflect a smaller decrease in MYC expression following SE1 disruption compared with SE2 due to the lower efficiency of sgRNA in disrupting the HLF motif within SE1 compared to SE2. Together, these results confirm *MYC* as target of TCF3::HLF and illustrate the added value of 3D genome information to study the TCF3::HLF-regulome.

### TCF3::HLF activation of MEF2C promotes expansion of leukemia cells

**Fig. 5.**
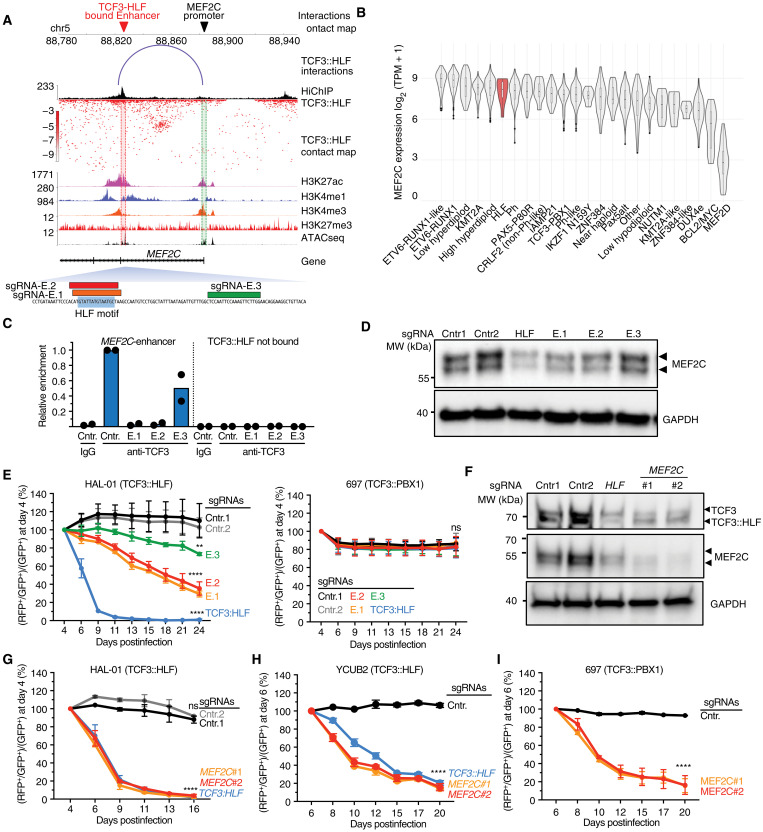
TCF3::HLF-mediated regulation of MEF2C promotes expansion of leukemia cells. (**A**) Visualization of the genomic interactions between MEF2C promoter and its enhancer, bound by TCF3::HLF, including TCF3::HLF-HiChIP, H3K27ac, H3K4me1, H3K4me3, and H3K27me3 ChIPseq, and ATACseq profiles. sgRNAs targeting the HLF motif at the *MEF2C*-enhancer and downstream regions are shown. (**B**) Violin plot of *MEF2C* expression across different ALL subtypes. Datasets from the St. Jude Cloud (*46*). (**C**) TCF3::HLF-ChIP-qPCR with TCF3 antibodies and Cas9-expressing HAL-01 cells with sgRNAs targeting the *MEF2C-*enhancer HLF motif (E.1 and E.2), downstream region (E.3), and sgRNA-Control (Cntr1). Measurements of TCF3::HLF association with *MEF2C*-enhancer and a desert region of Chr. 2 are shown. Values were normalized to input and TCF3:HLF enrichment at *MEF2C*-enhancer in cells treated with sgRNA-Control. (**D**) Western blot of *MEF2C* expression in Cas9-expressing HAL-01 cells transduced with sgRNA-Control and sgRNAs targeting *TCF3::HLF* (HLF) or the *MEF2C*-enhancer. GAPDH serves as loading control. (**E**) Competitive assay quantification in HAL-01 and 697 cells upon *MEF2C*-enhancer KO. Values correspond to RFP^+^/GFP^+^ and GFP^+^ cell number ratio at the indicated days relative to RFP^+^/GFP^+^ and GFP^+^ cell number ratio measured at day 4 posttransduction. Statistical significance (*P* values) of two independent experiments was calculated with two-way repeated measures ANOVA (**P < 0.01 and ****P < 0.0001). (**F**) Western blot showing MEF2C and TCF3::HLF expression in Cas9-expressing HAL-01 cells transduced with control or targeting sgRNAs. GAPDH serves as a loading control. (**G** to **I**) Competitive assay quantification in HAL-01 (G), YCUB-2 (H), and 697 (I) leukemia cells upon *MEF2C*-KO. Values correspond to the ratio between RFP^+^/GFP^+^ and GFP^+^ cell number at the indicated days relative to the ratio value at day 4 or 6 posttransduction. All results are mean values from two independent experiments. Statistical significance (*P* values) of two independent experiments was calculated with two-way repeated measures ANOVA (****P < 0.0001). MW, molecular weight.

The *MEF2C* gene ranks among the most significant E-P interaction through TCF3::HLF binding (Figs. 3F and 5A). Moreover, *MEF2C* is significantly down-regulated upon *TCF3::HLF*-KO in the parental HAL-01 cells, supporting the notion that *MEF2C* is directly activated by TCF3::HLF. *MEF2C* is expressed homogeneously in most ALL subtypes, including TCF3::HLF-positive ALL, and with the notable exception of MEF2D::BCL9 ALL, a rare ALL subtype with poor outcome (*52*–*54*) (Fig. 5B). MEF2C and MEF2D are evolutionarily conserved and important for the development of different tissues including blood (*55*). To determine the importance of MEF2C in TCF3::HLF-positive B-ALL as a direct target of TCF3::HLF, we disrupted the TCF3::HLF binding site within the *MEF2C* enhancer by CRISPR in HAL-01 cells and measured TCF3::HLF occupancy and MEF2C expression (Fig. 5, C and D, and fig. S4A). We performed TCF3::HLF-ChIP analysis in HAL-01 cells using anti-TCF3 antibodies (Fig. 5C). We found that TCF3::HLF association with the *MEF2C* enhancer was strongly reduced in cells treated with sgRNAs targeting the HLF motif compared to cells treated with sgRNA-Control or sgRNA-3 targeting a sequences downstream the HLF motif. These results not only further show that TCF3::HLF associates with the *MEF2C* enhancer but also that HLF sequences are necessary for TCF3::HLF binding. Last, the deletion of this HLF motif caused the down-regulation of MEF2C at RNA and protein levels, supporting a central role for this TCF3::HLF-bound enhancer for MEF2C regulation in TCF3::HLF-positive ALL (Fig. 5D and fig. S4A).

To determine the functional consequences of the association of TCF3::HLF with *MEF2C* enhancer, we performed a cell competitive assay using two TCF3 rearranged cell lines [697 with t(1;19) and HAL-01 t(17;19)] stably expressing Cas9-GFP (Fig. 5E and fig. S3B). The 697 ALL cell line harbors the TCF3::PBX1 fusion protein, which does not bind the HLF motif. Both cells were transduced with a lentiviral vector expressing RFP and sgRNAs targeting either *TCF3::HLF* or the TCF3::HLF-bound *MEF2C* enhancer site. Cell proliferation was quantified by flow cytometry by measuring the number of GFP^+^ and RFP^+^ cells from day 4 after infection until day 24. As expected, targeting *TCF3::HLF* in HAL-01 cells strongly impaired cell proliferation, whereas it had no effect in 697 cells (Fig. 5E), indicating the specificity of the assay and further supporting the essential role of TCF3::HLF in t(17;19)-positive ALL cells. Targeting the HLF site at the *MEF2C* enhancer also decreased cell proliferation in TCF3::HLF-positive ALL but not in TCF3::PBX1-positive ALL cells, suggesting that the HLF motif at this MEF2C enhancer plays an important and specific role in cell proliferation specifically for TCF3::HLF-positive ALL. Accordingly, the depletion of TCF3::PBX1 in 697 cells did not affect MEF2C expression levels, indicating that TCF3::PBX1 does not regulate MEF2C (fig. S4C). To further support the critical role of MEF2C in TCF3::HLF ALL cells, we performed the cell competitive assay in HAL-01 cells using sgRNAs targeting *MEF2C* exon 2 or 3 (*MEF2C#1 and #2*). Western blot analyses in HAL-01 cells confirmed MEF2C down-regulation (Fig. 5F). The reduction of MEF2C correlated with the down-regulation of TCF3::HLF, suggesting a regulatory loop between TCF3::HLF and MEF2C. Notably, MEF2C-KO impaired cell proliferation not only in HAL-01 cells but also in another TCF3::HLF t(17;19)-positive ALL cell line, YCUB-2, at a rate similar to *TCF3::HLF*-KO, underscoring the critical role of MEF2C (Fig. 5, G and H). Competitive assay with TCF3::HLF-positive patient-derived xenografts (PDXs) showed a notable dropout upon MEF2C-KO (fig. S4D). While the disruption of the MEF2C enhancer did not affect the proliferation of TCF3::PBX1-positive 697 cells, MEF2C-KO caused a marked reduction in cell proliferation (Fig. 5I). These findings indicate that MEF2C is essential for the growth of 697 cells, but its expression is not regulated by TCF3::PBX1. Collectively, these results indicate that the TCF3::HLF-mediated regulation of *MEF2C* through the interaction with the enhancer plays an important role in t(17;19)-positive ALL.

### Activation of MEF2C by TCF3::HLF induces a gene expression program that maintains HSC features

**Fig. 6.**
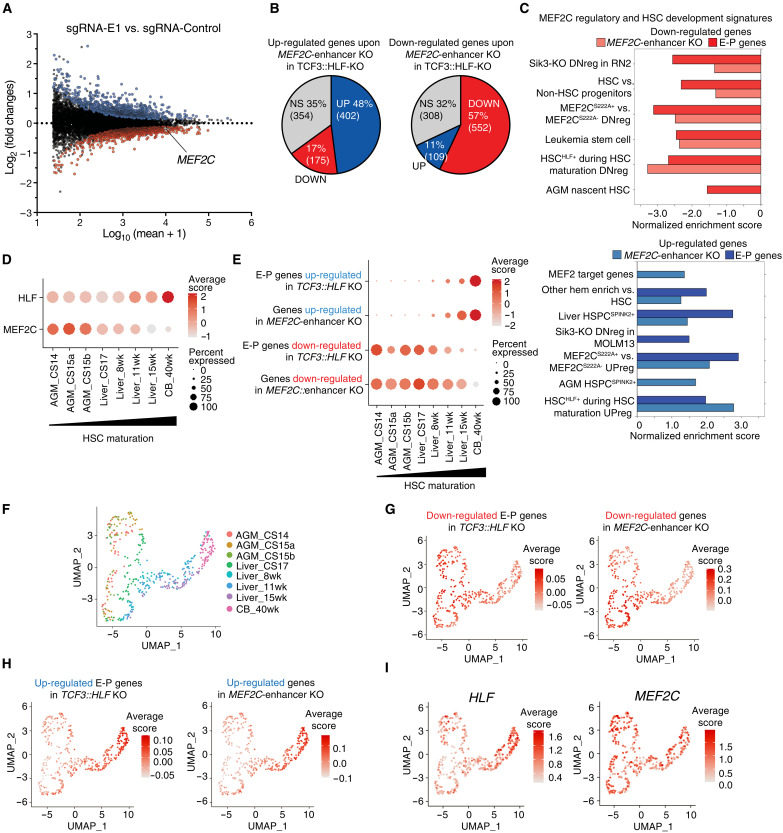
TCF3::HLF and MEF2C activity are required for maintenance of an early stem-like gene expression signature. (**A**) MA-plot showing differentially expressed genes upon MEF2C-enhancer KO in HAL-01 cells. Genes significantly up-regulated (adj. *P* < 0.05) are represented in blue, while down-regulated genes are in red. MEF2C gene is highlighted. (**B**) Pie charts showing the number of genes up-regulated or down-regulated upon MEF2C-enhancer KO that were regulated upon TCF3::HLF-KO in HAL-01 cells. (**C**) GSEA of up-regulated and down-regulated genes upon MEF2C-enhancer KO or *TCF3::HLF*-KO with MEF2C and HSC maturation gene sets from (*19*). The *x* axis displays the normalized enrichment score (NES) for significant values (adj. *P* < 0.05). Right panel displays positive NES values and left panel shows negative NES values. (**D**) Dot plot comparing the expression of HLF and MEF2C during HSC maturation. Data are from (*19*). (**E**) Dot plot comparing module scores of top 200 up-regulated and down-regulated genes (adj. *P* < 0.05) upon MEF2C-enhancer KO or of E-P genes that are up- and down-regulated upon *TCF3::HLF*-KO in HAL-01 cells. Data are from (*19*). Carnegie stages (CS)14/15 aorta-gonad-mesonephros (AGM), CS17 to 15 weeks liver, and 40 weeks cord blood (CB). The colour intensity and the size of the dot plots correspond to average score and percent expression, respectively. (**F**) Uniform Manifold Approximation and Projection (UMAP) analysis showing the clustering of scRNA-seq of HSC maturation. Data are from (*19*). (**G** and **H**) HSC maturation scorecard dot plot showing top 200 up-regulated (G) and down-regulated genes (H) upon MEF2C-enhancer KO or from E-P genes differentially expressed upon *TCF3::HLF*-KO in HAL-01 cells. (**I**) UMAP analysis highlighting MEF2C and HLF expression.

To establish whether and how TCF3::HLF-mediated regulation of MEF2C can have an impact on gene expression programs linked to disease, we analyzed the RNA-seq data of HAL-01 cells treated with sgRNA targeting the HLF motif of the *MEF2C* enhancer (*MEF2C*-enhancer KO). Cells were collected 13 days posttransduction, a time point at which HAL-01 exhibited approximately a 40% dropout rate in the competitive assay (Figs. 5E and 6A). We detected significant changes in gene expression (1021 up-regulated and 969 down-regulated genes, adj. *P* < 0.05), including, as expected, the down-regulation of *MEF2C* (Fig. 6A and table S8). Next, we compared the MEF2C-enhancer KO RNA-seq with the published RNA-seq profile of HAL-01 cells upon TCF3::HLF KO (Fig. 6B) (*20*). Among the genes up-regulated upon MEF2C-enhancer KO, 47% were also up-regulated upon TFC3::HLF-KO, whereas only 17% were down-regulated. Similarly, among genes down-regulated upon MEF2C-enhancer KO, 57% were likewise down-regulated upon TFC3::HLF-KO, while only 11% were up-regulated. Notably, genes co–up-regulated in both conditions were significantly enriched in processes linked to antigene processing, leukocyte and lymphocyte activation and differentiation, and hemopoiesis (table S8). These results indicate that TCF3::HLF, by regulating MEF2C expression through its association with MEF2C enhancer, blocks transcriptional programs that normally drive lymphoid differentiation. Gene set enrichment analysis (GSEA) with HALLMARK signatures showed that the *MEF2C*-enhancer perturbation caused the down-regulation of distinct gene expression programs, including *E2F* and *MYC* target genes (fig. S5A and table S8), in agreement with results using independent models showing that MEF2C modulates *E2F* and *MYC* expression (*30*). GSEA with curated gene sets of biological pathways revealed positive enrichment of a B cell progenitor signature after perturbation of the *MEF2C* enhancer, in addition to negative enrichment of genes involved in cell proliferation, pediatric cancers, and stemness (fig. S4B and table S8). GSEA using a custom collection of published signatures from after *MEF2C*-KO or perturbed MEF2C activity (*30*, *56*, *57*) revealed overlapping signatures between genes down-regulated upon *MEF2C*-enhancer KO and *TCF3::HLF*-KO and down-regulated genes after MEF2C inactivation (Fig. 6C and table S8).

Since HLF is one of the most specific TFs identifying human HSCs, we investigated gene sets of human HSC identity and maturation throughout development and aberrantly self-renewing progenitor-like cancer stem signature (*19*, *58*). *MEF2C-*enhancer KO expression profiles showed the enrichment of signatures of both hematopoietic differentiation and HSC maturation (e.g., HSC^HLF+^ during HSC maturation UPreg) for up-regulated gene targets, whereas signatures of undifferentiated HSC identity and immature nascent HSC were negatively enriched (e.g., HSC^HLF+^ during HSC maturation DNreg, HSC versus non-HSC progenitors). Leukemia stem cell signatures were also negatively enriched (Fig. 6C). These results suggest that MEF2C is a main driver of stemness programs in this TCF3::HLF-positive leukemia. Furthermore, the same programs of HSC maturation and differentiation were also enriched in the HiChIP-filtered dataset including TCF3::HLF-bound E-P loop genes that are regulated after *TCF3::HLF*-KO (Fig. 6C and table S9).

We further investigated how genes modulated upon *TCF3::HLF*-KO and *MEF2C*-enhancer KO are regulated throughout HSC maturation by interrogating publicly available single-cell RNA-seq (scRNA-seq) datasets mapping the transcriptomic changes in human embryonic HLF^+^ HSCs throughout maturation: from the aorta-gonad-mesonephros [AGM; Carnegie stage (CS) 14/15] region, the transition to the fetal liver until week 15, to cord blood (CB) (*19*). We found that HLF expression increased with HSC maturation, whereas MEF2C expression was highest in the most immature embryonal subsets (AGM HSC) (Fig. 6D). The module score analysis of the differentially expressed genes after *TCF3::HLF*-KO with E-P contacts, as well as differentially expressed genes after *MEF2C*-enhancer KO, revealed a pattern concordant with the expression of MEF2C throughout maturation: Up-regulated genes are mostly expressed in mature HSC (i.e., 15 week fetal liver and CB), while down-regulated genes are preferentially expressed in immature HSCs (i.e., AGM, early liver) (Fig. 6, E to I).

To determine how TCF3::HLF and MEF2C target genes are regulated throughout hematopoietic differentiation, we analyzed a published single-cell transcriptome map of HSCs and differentiated progenitors from human bone marrow (*59*). These data represent mature HSC, multipotent progenitors, megakaryocyte-erythroid progenitors, common myeloid progenitors (CMPs) and granulocyte-monocyte progenitors (GMPs), multi-lymphoid progenitors (MLPs), Pre-B lymphocytes/natural killer (Pre-B/NK) cells. *MEF2C* was expressed in lymphoid progenitor subsets (Pre-B/NK and MLP) compared to the more undifferentiated subsets, while HLF expression was almost exclusive to HSCs (fig. S5C). Module score analysis revealed that E-P genes up-regulated after *TCF3::HLF*-KO and up-regulated genes after *MEF2C*-enhancer KO are normally expressed in lymphoid/B progenitors, consistent with signatures observed in TCF3::HLF leukemia cells (*20*). Down-regulated genes were more expressed in less differentiated progenitors such as CMP and GMP (fig. S5, D to H). Collectively, these results suggest that TCF3::HLF and its regulation of *MEF2C* via enhancer binding contribute to driving programs associated with immature HSC and preventing the activation of lymphoid programs in leukemia.

## DISCUSSION

In this study, we have delineated the role of the fusion protein TCF3::HLF in the 3D genomic landscape to promote leukemogenesis in t(17;19)-positive ALL. The integration of HiChIP with RNA-seq and modified histone ChIPseq data has revealed key E-P interactions mediated by TCF3::HLF, providing a comprehensive view of the gene regulatory networks underlying this aggressive leukemia subtype. Although our analysis in this study was limited to interaction pairs containing HLF motifs, reflecting the location of the DBD of TCF3::HLF in the HLF region, the HiChIP data generated will serve as a valuable resource for identifying additional noncanonical TCF3::HLF genomic loops and characterizing their functional roles in t(17;19)-positive ALL.

This interaction map provides new insights into genes that are both positively and negatively regulated by the oncogenic fusion. Simultaneous direct repression by the oncogenic TF must contribute to canalizing the abnormal cell fate, as suggested by many examples for fate decision through repression of genes relevant for differentiation (*60*). Our previous TCF3::HLF interactome analysis identified potential cofactors that can be recruited to alter chromatin states, such as members of switch–sucrose nonfermentable (SWI-SNF) chromatin remodeling complex (SMARCA2, SMARCC1, and SMARCD2) and mediator factors promoting DNA loops interactions (YY1, CTCF, CGGBP1, and ZNF512) (*20*).

Specific E-P activity renders TCF3::HLF highly dependent on MYC. Besides sustaining metabolic states to favor proliferation, MYC can regulate chromatin decompaction in cells with high plasticity including activated B cells (*61*). We identify additional functionally relevant HLF binding sites within the BENC that are critical determinants for leukemia propagation. HLF is established as one of the most specific HSC identity genes. HLF is expressed in naïve stems cells and multipotent hematopoietic progenitors (*17*, *18*, *62*) and in fetal HSCs (*19*, *63*). TCF3::HLF must reprogram a cell of origine to an immature lineage ambivalent state. Here we show that TCF3::HLF regulates *MEF2C* directly, in one of the most significant E-P loop detected by HiChIP in leukemia cells. MEF2C has been implicated as a regulator of the development in various tissues including of lymphoid T, B, and NK lineages (*31*, *35*, *37*, *55*). MEF2C is also activated as an oncogene by structural rearrangements in T-ALL, deregulation through leukemic TFs (e.g., GFI1B and NKX2-5), and loss of inhibitory control through signal transducers and activators of transcription 5 signaling (*64*). TCF3::HLF is also dependent on BCL2, similar to more immature lymphoid subtypes (*65*) and leukemic stem cells in acute myeloid leukemia (*66*). Here, we identify the critical HLF binding site and enhancer that activates BCL2, which represents a relevant drug target in TCF3::HLF (*21*) that translated in molecular responses to venetoclax in combination with chemotherapy in compassionate treatment attempts and to the systematic recommendation for this leukemia subtype by the AIEOP-BFM Study Group in Europe (NCT03643276). Moreover, we show that the perturbation of specific TCF3::HLF binding at the MEF2C enhancer did not only attenuate leukemia proliferation but also altered transcription to activate the expression of genes associated with HSC maturation. We detect MEF2C as a component of primitive HSC gene expression signatures in fetal HSCs and adult stem and progenitor cells. The functional identification of MEF2C as a critical target of TCF3::HLF emphasizes the importance of specific E-P interactions in the maintenance of leukemic proliferation and stem-like characteristics.

Together, our results highlight fundamental components of the transcriptional circuitry that is rewired by this oncogenic fusion protein to drive the malignant phenotype. This reinforces the central need to develop new tools to target these fundamental driver mechanisms. This map of E-P interactions constitutes an important resource for future functional exploration of intragenic and extragenic TCF3::HLF-mediated functions. A deep understanding of oncogenic programs will be required to pave the way for more effective combinations of targeted treatments for patients suffering from these devastating diseases.

## MATERIALS AND METHODS

### Cell lines and cell culture

The human leukemia cell lines used in this study were HAL-01 (Leibniz Institute, DSMZ, ACC 610) (*12*), YCUB-2 (Leibniz Institute, DSMZ, ACC 961), and 697 (Leibniz Institute, DSMZ, ACC 42). HAL-01 and 697 cell lines were purchased from DSMZ. YCUB-2 was a generous gift from DSMZ. Leukemia cell lines were not further authenticated upon receipt, but the presence of the expected TCF3-fusion was validated by RNA-seq and Western blotting. All leukemia cell lines were grown in RPMI 1634 Media (Gibco) supplemented with 10% fetal bovine serum (FBS), 1% glutamine (Gibco), penicillin (100 U/ml), and streptomycin (100 μg/ml). Human telomerase reverse transcriptase (hTERT)–immortalized human bone marrow mesenchymal stromal cells (MSCs) were grown in RPMI 1640 supplemented with 10% FBS (Merck, #S0615), 1% GlutaMAX (Gibco, #35050-038), 1% penicillin-streptomycin (Thermo Fisher Scientific, #15140122), and 1 μM hydrocortisone (Sigma-Aldrich, #R0883). Lenti-X 293T cells (Takara, 632180) used for virus production were grown in DMEM (Thermo Fisher Scientific, #11960044) supplemented with 10% FBS, 2% GlutaMAX, and 1% penicillin-streptomycin. TCF3::HLF PDX cells were grown in Plasmax medium (CancerTools, #156371) supplemented with 2.5% FBS and 1% penicillin-streptomycin. All cells were cultured in a 37°C 5% CO_2_-humidified incubator and routinely checked for mycoplasma using the LookOut Mycoplasma PCR Detection Kit (Sigma-Aldrich, MP0035-1KT).

### Establishment of the stable TCF3-KO HAL-01 line

To establish TCF3-KO HAL-01 cell line, lentiviral transductions were performed as previously described (*20*). HAL-01_Cas9 cells were transduced with sgRNA targeting TCF3. Following transduction, cells were diluted in supplemented RPMI medium to a concentration of 20 cells/ml and seeded into a 384-well flat-bottom plate. Once cultures reached ~25% confluency, cells were sequentially transferred to 24-well and then 12-well plates upon reaching the same confluency threshold at each step. Resulting colonies were analyzed by flow cytometry and Western blot to confirm clonal expansion and successful knockout of wild-type TCF3.

### Patient sample and PDX

The TCF3::HLF-positive patient sample was collected within the International BFM Study Group (I-BFM-SG) as described previously (*21*). Informed consent was given in accordance with the Declaration of Helsinki. Approval for experiments with human samples in the mouse xenograft model was obtained from the ethics commission of the Canton Zurich (approval number 2014-0383). For generating PDXs, primary human ALL cells were transplanted into 5- to 12-week-old immunodeficient NOD/SCID/IL2rγnull (NSG) mice via tail vein injection (*67*). Mice were purchased from the Jackson Laboratory (RRID: IMSR_JAX:005557), housed, and bred under pathogen-free conditions in our local animal facility (University of Zurich). All animal experiments were approved by the veterinary office of the Canton of Zurich, Switzerland. Leukemia progression was monitored weekly by flow cytometry (Fortessa LSR, BD bioscience) of peripheral blood using anti (α)–human CD19–phycoerythrin (BioLegend, #302208), α–hCD45–Alexa Fluor 647 (BioLegend, #304018), and α–mCD45–eFluor 450 (Invitrogen, #48-0451-82). ALL cells harvested from spleens of NSG mice were used for competitive assays experiments.

### Lentivirus production

Lentiviral particles were generated by transient transfection of Lenti-X 293T cells with the transfer plasmid, psPAX2 (Addgene, #12260), and pCMV-VSV-G (Addgene, #8454) at a ratio of 5:3:2 using XtremeGene 9 transfection reagent (Roche, catalog no. XTG9-RO), following the manufacturer’s instructions. Viral supernatants were collected after 30 hours, centrifuged at 350*g*, and filtered through a 0.45-μm membrane to remove cell debris. For CRISPR-Cas9–mediated KOs, the virus was subsequently concentrated using Amicon Ultra-15 centrifugal filter units with a 100–kDa molecular weight cutoff (Millipore, UFC910024).

### shRNA-mediated knockdown of TCF3::PBX1

The short hairpin RNA (shRNA)–mediated knockdown was achieved using the tetracycline-inducible vector pRSIT-U6Tet-shTarget-PGK-TetRep-2A-TagGFP2-2A-Puro (Cellecta). The shRNA sequence targeting the PBX1 C-terminal region of TCF3::PBX1 and the scrambled control shRNA were generated by annealing complementary oligonucleotides (Microsynth) and ligating them into the Bbs I–linearized backbone (Thermo Fisher Scientific, ER1011). The sequence of the shRNAs used is: CAACAAGATGAAGAGCACCAA (shSCR) and GGAGCATTCAGATTACAGA (shPBX1). To generate dox-inducible shRNA lines, 697 cells were transduced with crude viral supernatant at a 1:1 ratio with fresh medium for 48 hours, followed by washing with phosphate-buffered saline (PBS). Successfully transduced cells were enriched by fluorescence-activated cell sorting (FACS) based on green fluorescent protein expression. shRNA expression was induced in transduced cells by supplementing the culture medium with doxycycline (100 ng/ml; Sigma-Aldrich, D981-10G) every 2 to 3 days.

### Western blotting

Whole-cell lysates were prepared by resuspending cell pellets in Laemmli (Bio-Rad, #1610747) buffer with beta-mercaptoethanol and boiling for 6 to 10 min. Protein electrophoresis was performed using 4 to 20% Mini-PROTEAN TGX Precast Protein Gels (Bio-Rad) or 4 to 12% Criterion XT Bis-Tris Gel (Bio-Rad). The proteins were transferred to Trans-Blot Turbo Midi 0.2 μm Nitrocellulose (Bio-Rad) and immunoblotted using following antibodies: E2A (D2B1) Rabbit mAb (Cell Signaling Technology, catalog no. 12258, RRID:AB_2797860), c-Myc (D84C12) Rabbit mAb (Cell Signaling Technology, catalog no. 5605, RRID:AB_1903938), MEF2C (D80C1) Rabbit mAb (Cell Signaling Technology, catalog no. 5030, RRID:AB_10548759), glyceraldehyde-3-phosphate dehydrogenase (D16H11) Rabbit mAb (Cell Signaling Technology, catalog no. 5174, RRID:AB_10622025), Anti-rabbit immunoglobulin IgG (IgG)–horseradish peroxidase (Sigma-Aldrich, catalog no. A0545, RRID:AB_257896). Signal development was done with SuperSignal West Pico PLUS [Thermo Fisher Scientific; (*21*)] ECL reagent in a Chemidoc Touch Imaging System (Bio-Rad).

### Competitive assays

Lentiviral transductions were performed as previously described (*20*). Cell lines and TCF3::HLF^+^ PDX cells stably expressing Cas9 and a GFP reporter were previously established using the PX458 plasmid backbone (Addgene, catalog no.48138) (*68*). sgRNAs were cloned into the shuttle plasmid coexpressing the RFP657 reporter (Addgene, catalog no. 134968) (*20*), as previously described. Efficiencies were determined by flow cytometry on day 3 after transduction. For the competitive assays, Cas9-GFP–expressing cells were transduced with sgRNA-RFP and mixed 1:1 with the same untransduced cell lines. PDX cells were transduced with sgRNA-RFP in monoculture, and 24 hours after viral transduction and washout, they were plated onto MSCs. Starting from day 4 after transduction, the distribution of the (GFP/RFP) and mono-(GPF) fluorescent populations were analyzed by flow cytometry sequentially. At each annotated time point, the ratio of double-positive cells was calculated in reference to baseline at day 4.

### Quantification of MYC-SE1/2 KO efficiency

RNA-free genomic DNA was isolated from HAL-01-Cas9 cells harvested 6 days after transduction with sgRNA-expressing vectors using the DNeasy Blood & Tissue Kit (QIAGEN, 69504) in combination with ribonuclease A (Thermo Fisher Scientific, EN0531). The efficiency of the sgRNAs in disrupting the HLF motif was assessed by quantitative polymerase chain reaction (qPCR) using the PowerUp SYBR Green Master Mix (Applied Biosystems, A25742). For each reaction, 5 ng of genomic DNA was used in a 384-well plate format, and amplification was performed on a QuantStudio 7 Pro Real-Time PCR System (Applied Biosystems). The fold enrichment of MYC-SE1 and MYC-SE2 regions was calculated relative to a control genomic region (desert) and subsequently normalized to the nontargeting control (sgRNA-Control) condition. Data are from two to three biological replicates, each including four technical replicates. Primers were designed using NCBI Primer-BLAST and synthesized by Microsynth and described in table S10. Forward primers for MYC-SE1 and MYC-SE2 anneal upstream of the target site (HLF motif), whereas reverse primers partially overlap with the downstream part of the target site, as done previously (*20*).

### ChIP and HiChIP sample processing

ChIP and HiChIP chromatin sample preparation followed the MNase HiChIP Dovetail Kit (Cantata Bio) protocol v1.3 with modifications. Briefly, cells were harvested in batches of 30 million cells, pelleted, and frozen at −80°C for at least 30 min before fixation. Pellets were thawed on ice and washed once in PBS. Double cross-linking was performed using 1.7 mM ethylene glycol disuccinate di(*N*-succinimidyl) ester (EGS; Thermo Fisher Scientific, #21656) in PBS for 45 min at room temperature, followed by 10 min in 1% formaldehyde (v/v) in PBS. Formaldehyde was quenched by adding glycine to a final concentration of 125 mM. Fixated cells were pelleted, snap frozen, and stored at −80°C until further use.

Fixed frozen cell pellets were allowed to thaw at room temperature and washed in 1× Dovetail wash buffer according to protocol and resuspended in micrococcal nuclease (MNase) digestion buffer using 100 μl of buffer per 10 million cells. 1000 U of MNase was added per 100 μl of buffer and incubated for 6 min for chromatin digestion. The reaction was quenched by adding 50 mM EGTA. Samples were diluted in MNase digestion buffer and supplemented with SDS to a final concentration of 0.1% and divided into two sonication vials, 15 million cells in 400 μl of MNase digestion buffer each. Using maxed power settings eight cycles with 30-s on and 30-s off, the chromatin was released from the cells (Micro Pulse Digenode). The chromatin fragmentation quality was validated with Agilent D5000HS ScreenTape System for DNA on a 2200 TapeStation platform, only digestions with a distribution of mononucleosomes between 40 and 70% were used for HiChIP.

For both ChIP and HiChIP, 15 μg of chromatin sample was used for pulldown with TCF3 antibodies (Cell Signaling Technology, catalog no. 12258, RRID:AB_2797860). The chromatin was incubated overnight with A/G magnetic Dynabeads coated with E2A (D2B1) Rabbit mAb (Cell Signaling Technology, catalog no. 12258, RRID:AB_2797860) or Rabbit IgG Isotype control (Cell Signaling Technology, catalog no. 3900, RRID:AB_1550038). Magnetic beads were washed with increasing stringencies of salt buffers with 150 mM NaCl, 500 mM NaCl, 250 mM LiCl, and lastly TE buffer according to ChIP Abcam protocols, (Dovetail washing procedure was not followed). For ChIP-qPCR, the samples were eluted at this stage by using the Dovetail Kit 1xReversal of crosslinks salt buffer and proteinase K incubated at 55° for 15 min and 68° for 45 min and purified using the PCR Purification Kit from QIAGEN. Primers used for qPCR measurements are listed in table S10.

For HiChIP the proximity ligation protocol was followed as instructed by the manufacturer. After streptavidin purification, the library preparation was done with the Dovetail Library prep module according to the manufacturer’s protocol. The PCR amplification was done as recommended for the amount of pulled-down DNA material. Generated libraries were filtered by size using SpriSelect beads (Beckman Coulter). HAL-01 HiChIP biological replicates were pooled and sequenced using NovaSeq 6000 with 400 million reads and 2 × 150-base pair (bp) paired-end.

### HiChIP analysis

The HiChIP analysis pipeline documented in the HiChIP documentation release 0.1 by Dovetail (https://hichip.readthedocs.io/en/latest/index.html) was followed with some modifications. HiChIP paired-end read files were trimmed using TrimGalore (v0.6.6) with Phred+33 quality encoding. Reads shorter than 50 bp or with base quality scores below a Phred cutoff of 20 were removed. Biological replicates were merged as recommended. HiChIP reads were aligned to the hg38 genome using Burrows-Wheeler Aligner (*69*) using the BWA-MEM algorithm with settings as described in the HiChIP documentation release 0.1. Interaction events are extracted with the parse module of pairtools, followed by sorting and splitting of the generated pairsam file into mapped pairs and mapped bam files, and the pairtools dedup step was omitted. We used mapped bam files to generate primary aligned bed files for 1D MACS2 (*70*) peak calling as reference when calling genomic interactions with FitHiChIP (*24*) using a bin size of 5 kb and range settings of 50 kb to 3 Mb.

For the summary of the stratum-adjusted correlation coefficient (SCC) between the TCF3 HiChIP replicates across all chromosomes, both contact .hic files generated for TCF3 HiChIP pulldown replicates were converted to .cool contact matrices at 100-kb resolution with hic2cool v0.8.3 (https://github.com/4dn-dcic/hic2cool). The generated cool files were used as input for R package hicrep (v1.12.2) to calculate the genome-wide reproducibility score, SCC, between corresponding chromosomes. Settings used for smoothing parameter *h* = 3 and lower and upper bound for genomic distances were set to 0 and 3,000,000, respectively. Scores for replicates that are >0.8 are acceptable, although lower values of 0.7 to 0.8 can be expected for smaller chromosomes or if structural variations are present.

### Peak and motif analysis

We integrated published histone ChIPseq data for HAL-01 in combination with HOMER v.4.7, peakAnnotation (*71*), annotation of enhancer regions using both the ROSE output of MACS2 called broad peaks from H3K27ac ChIPseq data described above and further verified with Fantom5 Enhancer database (table S1). Binding sites with a HLF motif were identified with the aid of the Simple enrichment analysis and Find Individual Motif Occurrences tools, from the MEME-suit (*72*), on MACS2 called peaks from TCF3::HLF HiChIP enrichment. Going by a ±10-kb window around transcription start sites (TSS) and integration of differential gene expression data from HAL-01 RNA-seq after TCF3::HLF-KO, we defined putative promoter anchor sites associated with TCF3::HLF-bound anchors.

### Histone ChIPseq and ATACseq data processing

The histone mark ChIPseq datasets for HAL-01 were downloaded from the European Nucleotide archive repository (ERP109232). Reads were trimmed using TrimGalore (v0.6.6) and aligned to the hg38 reference genome using BWA-MEM (*69*). BigWig files were generated with deeptools (*73*). We selected regions of interest from HiChIP TCF3::HLF identified sites with *HLF* motif and included top 75% of the regions with highest TCF3::HLF enrichment. Using deeptools computeMatrix analysis, we proceeded to define the histone coverage using our own data (*20*) and publicly available ATACseq data, GSE186942 (*74*), at TCF3::HLF-bound regions identified with HiChIP. Based on histone features, we performed clustering with computeMatrix into three main cluster groups.

### Calling enhancer clusters with ROSE

Enhancer clusters were called from H3K27ac ChIPseq data (*20*) as before using ROSE (*26*, *27*) on MACS2 broad peak calling outputs under the hg38 reference genome. Default parameters were chosen. Briefly, H3K27ac peaks were first clustered on basis of gap distance, and enhancer clusters were identified by strong H3K27ac ChIPseq enrichment. Together with the Fantom5 database (*28*, *29*), annotations of TCF3::HLF-associated enhancer sites were made (table S1). Interactions originating from annotated enhancers and interacting with specified E-P gene targets are summarized in table S5.

### TCF3-ChIPseq

ChIP analysis was performed as previously described (*75*). Briefly, 1% formaldehyde was added to TCF3-KO HAL-01 cells and parental HAL-01 cells to crosslink proteins to DNA. Formaldehyde was quenched by adding glycine to a final concentration of 125 mM. Fixated cells were pelleted, snap frozen, and stored at −80°C until further use. Isolated nuclei were then lysed with lysis buffer [50 mM tris-HCl (pH 8.1), 10 mM EDTA (pH 8), 1% SDS, and 1X protease inhibitor complete EDTA-free cocktail, Roche]. Nuclei were resuspended in MNase digestion buffer using 100 μl of buffer per 10 million cells. A 10 U of MNase was added per 100 μl of buffer and incubated for 30 min for chromatin digestion. The reaction was quenched by adding 50 mM EGTA. Samples were diluted in MNase digestion buffer and supplemented with SDS to a final concentration of 0.1% and divided into two sonication vials 15 million cells in 400 μl of MNase digestion buffer each. Using maxed power settings eight cycles with 30-s on and 30-s off, the chromatin was released from the cells (Micro Pulse Digenode), resulting in sheared genomic DNA of an average fragment size of 200 bp. A 100 μg of chromatin was diluted to a total volume of 500 μl with ChIP buffer [16.7 mM tris-HCl (pH 8.1), 167 mM NaCl, 1.2 mM EDTA, 0.01% SDS, and 1.1% Triton X-100] and precleared with 10 μl of packed Sepharose beads for 2 hours at 4°C. Precleared chromatin was incubated overnight with ChIP-grade TCF3 antibody (Cell Signaling Technology, #12258). The next day, Protein A Dynabeads (Invitrogen, 10002D) were added and incubated for 4 hours at 4°C. After washing, bound chromatin was eluted with the elution buffer (1% SDS and 100 mM NaHCO_3_). Upon proteinase K digestion (50°C for 3 hours) and reversion of cross-linking (65°C, overnight), the DNA was purified with phenol/chloroform, and precipitated with ethanol. The quantity and quality of the isolated DNA were determined with a Qubit 4 Fluorometer (Life Technologies, California, USA) and Agilent D5000HS ScreenTape System for DNA on a 4200 TapeStation platform. Briefly, ChIP samples (1 ng) were end-repaired and polyadenylated before the ligation of Illumina compatible adapters. The adapters contain the index for multiplexing. The quality and quantity of the enriched libraries were validated using Qubit 4 Fluorometer and the 4200 TapeStation system. The libraries were pooled to 10 nM in Tris-HCl 10 mM (pH 8.5) with 0.1% Tween 20. Sequencing was performed on the NovaSeqX Plus platform for 150-bp paired-end reads (Illumina, Inc., California, USA).

ChIPseq reads were aligned to the hg38 reference genome with Bowtie2 (*76*) (version 2.5.0) using default parameters. PCR duplicates were removed using samtools (*77*). Bigwig files were created from the bam files using bamCoverage from deeptools (*78*) using default parameters. Integrative Genome Viewer (*79*) (version 2.15.4) was used to visualize and extract representative ChIPseq tracks. TCF3 enriched peaks were called using MACS2 (version 2.2.7.1) (*70*) with the corresponding inputs as control. All heatmaps and profiles were generated using computeMatrix from deeptools (*78*).

### RNA-seq sample preparation

Total RNA samples were isolated using the Direct-zol RNA Miniprep with on column deoxyribonuclease digestion after lysis in TRI Reagent RT (Molecular Research Center). RNA-seq libraries were generated with the Truseq RNA Library Prep Kit for Illumina per the manufacturer’s instructions and sequenced on a NovaSeq 6000 with paired end of 2 × 100 bp.

### RNA-seq expression and GSEA analysis

RNA-seq reads were processed using Nextflow 23.04.4 nf-core/rnaseq 3.12.0 pipeline. Briefly, sequenced reads were trimmed for adapter sequences/low-quality reads with trimmgalore 0.6.7 (parameters --phred33 --length 30 -j 4). Trimmed sequences were aligned to the hg38 genome (GCA_000001405.15_GRCh38_no_alt_analysis_set) using STAR alignment 2.7.9a. The read count quantification was done with Salmon 1.10.1. The same analysis pipeline was used to reanalyze the *TCF3::HLF*-KO dataset from the European Nucleotide archive under accession number ERP109232. Normalization and differential analysis were performed using R/Bioconductor version 4.3.1 software package Deseq2 in RStudio. GSEA was done with the R package fgsea. The gene signatures used included HALLMARKS and curated gene sets of biological pathways (C2.signatures) (www.gsea-msigdb.org) and custom gene signature for MEF2C and HSC gene sets. All the gene set sources are listed in table S9. We performed a stratification of the St. Jude’s data by retrieving any genes with 3× higher median expression in TCF3::HLF-positive leukemias than all other subtypes. Data are from the St. Jude Cloud datasets (www.stjude.cloud) (*46*).

### scRNA-seq dataset comparisons

The expression of HLF and MEF2C were assessed in publicly available scRNA-seq datasets from (*19*) (human HSC maturation throughout development) and (*59*) (human bone marrow HSC and differentiated populations) using the R package Seurat (v3.1.2). Gene modules were compiled for the top 200 up-regulated and down-regulated genes (adj. *P* value > 0.05) after MEF2C-enhancer KO or after TCF3::HLF-KO, and selected for E-P genes and scores were calculated using AddModuleScore with default parameters. The module scores and the expression patterns of selected genes are shown using the DotPlot and FeaturePlot functions.

### Data visualization

RNA-seq expression and GSEA analysis were visualized with plots generated using R/Bioconductor version 4.3.1 software package ggplot2 (https://ggplot2.tidyverse.org). HiChIP heatmaps were generated using HiC-Pro by merging valid pairs of replicates and processed as .hic files using juicer tools v1.22.01 function and visualized using Juicebox tools. Coolbox API with Jupyter Notebook was used to summarize and generate genome visualizations for ChIPseq, ATACseq, and HiChIPseq data. Circular plots were generated using R package circlize v0.4.16. Plots for PCR outputs and competitive assay were generated using GraphPad Prism version 10.2.0 for Windows, GraphPad Software, Boston, MA, USA (www.graphpad.com).

### Statistical analysis

GraphPad Prism 10 was used to perform statistical comparisons. Statistical analyses between two groups were performed by using the unpaired *t* test. Statistical analyses among more than two groups was calculated either with two-tailed *t* test, followed by Benjamini-Hochberg correction or two-way repeated measures analysis of variance (ANOVA). *P* values <0.05 were considered statistically significant. Experimental data are presented as means ± SD.
