# Supplementary material for: TCF3::HLF orchestrates an enhancer-promoter network with activation of MEF2C to promote immature HSC gene expression in leukemia
Source: Sci Adv. 2026 May 6;12(19):eadu3728. doi: 10.1126/sciadv.adu3728 (PMC13148343; doi:10.1126/sciadv.adu3728)
Supplement: Supplementary file 1 — Figs. S1 to S5 Legends for tables S1 to S10 [file sciadv.adu3728_sm.pdf]

Science Advances
AAAS

Supplementary Materials for

TCF3::HLF orchestrates an enhancer-promoter network with activation of MEF2C to promote immature HSC gene expression in leukemia

Valdemar Priebe *et al.*

Corresponding author: Raffaella Santoro, raffaella.santoro@dmmd.uzh.ch;
Jean-Pierre Bourquin, jean-pierre.bourquin@kispi.uzh.ch

Sci. Adv. **12**, eadu3728 (2026)
DOI: 10.1126/sciadv.adu3728

The PDF file includes:

Figs. S1 to S5
Legends for tables S1 to S10

Other Supplementary Material for this manuscript includes the following:

Tables S1 to S10

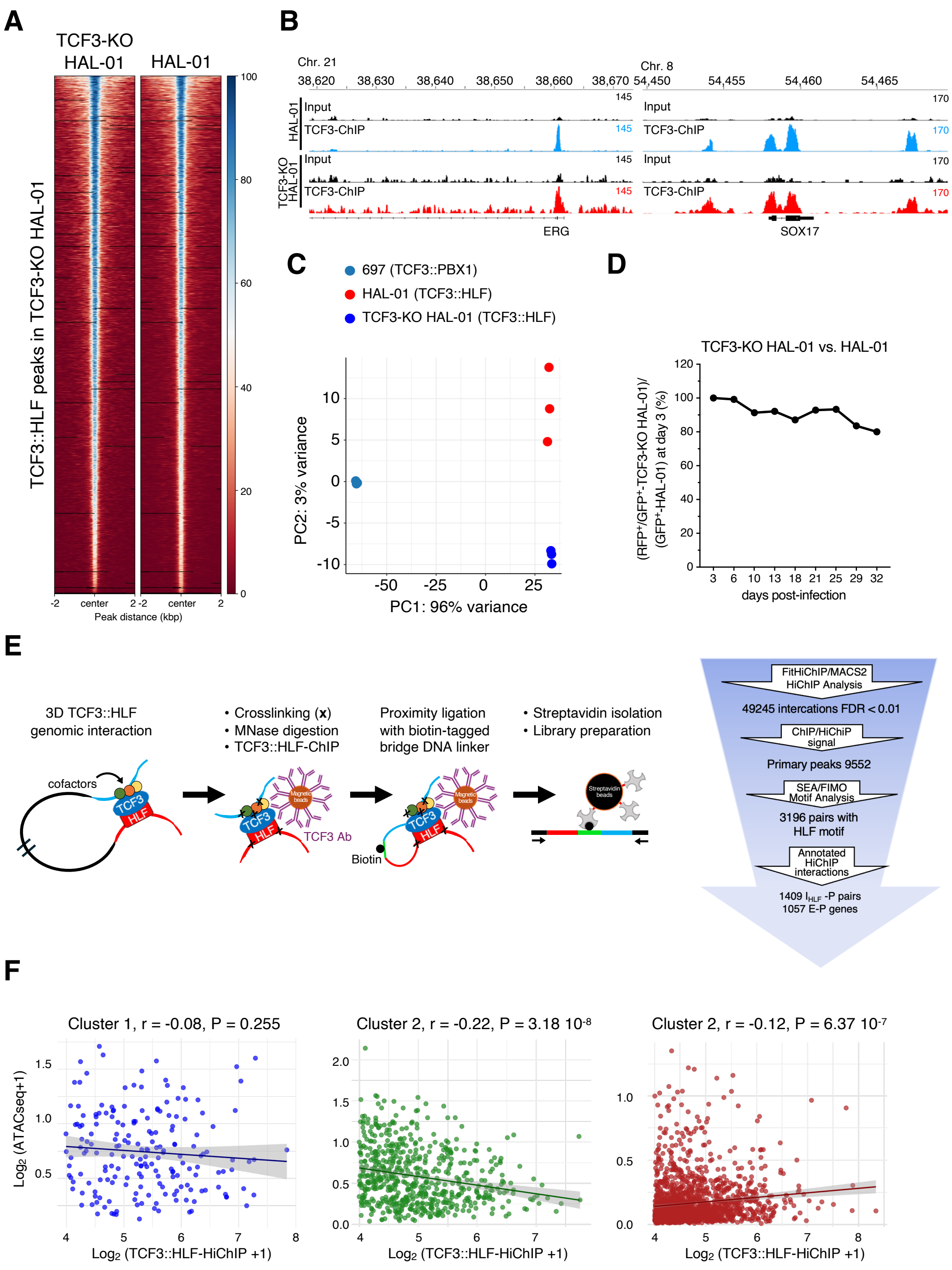

Figure S1. Characterization of TCF3-KO HAL-01 cell line and TCF3::HLF-HiChIP pipeline

(**A**) TCF3-ChIPseq of TCF3::KO HAL-01 cells and the parental HAL-01 cell line. Heatmap on TCF3::HLF peaks.

(**B**) Tracks showing TCF3-ChIPseq profiles at known TCF3-HLF binding sites

(**C**) Principal component analysis (PCA) of RNAseq data of TCF3-KO HAL-01 and parental HAL-01 cells and 697 cells containing TCF3::PBX1 fusion.

(**D**) Competitive assay quantification between GFP+-RFP+ TCF3::KO HAL-01 cells and the parental GFP+ HAL-01 cell line. Values correspond to the ratio between RFP+/GFP+ (i.e.

+sgRNA) and GFP+ (no sgRNA) cell number at the indicated days relative to the ratio between RFP+/GFP+ and GFP+ cell number measured at day 3 post-transduction.
(**E**) Schematic summary of the HiChIP strategy used to identify pairs of genomic interactions bound by TCF3::HLF and downstream analysis.
(**F**) Scatter plots of ATACseq and TCF3:HLF-ChIPseq of clusters 1, 2, and 3.

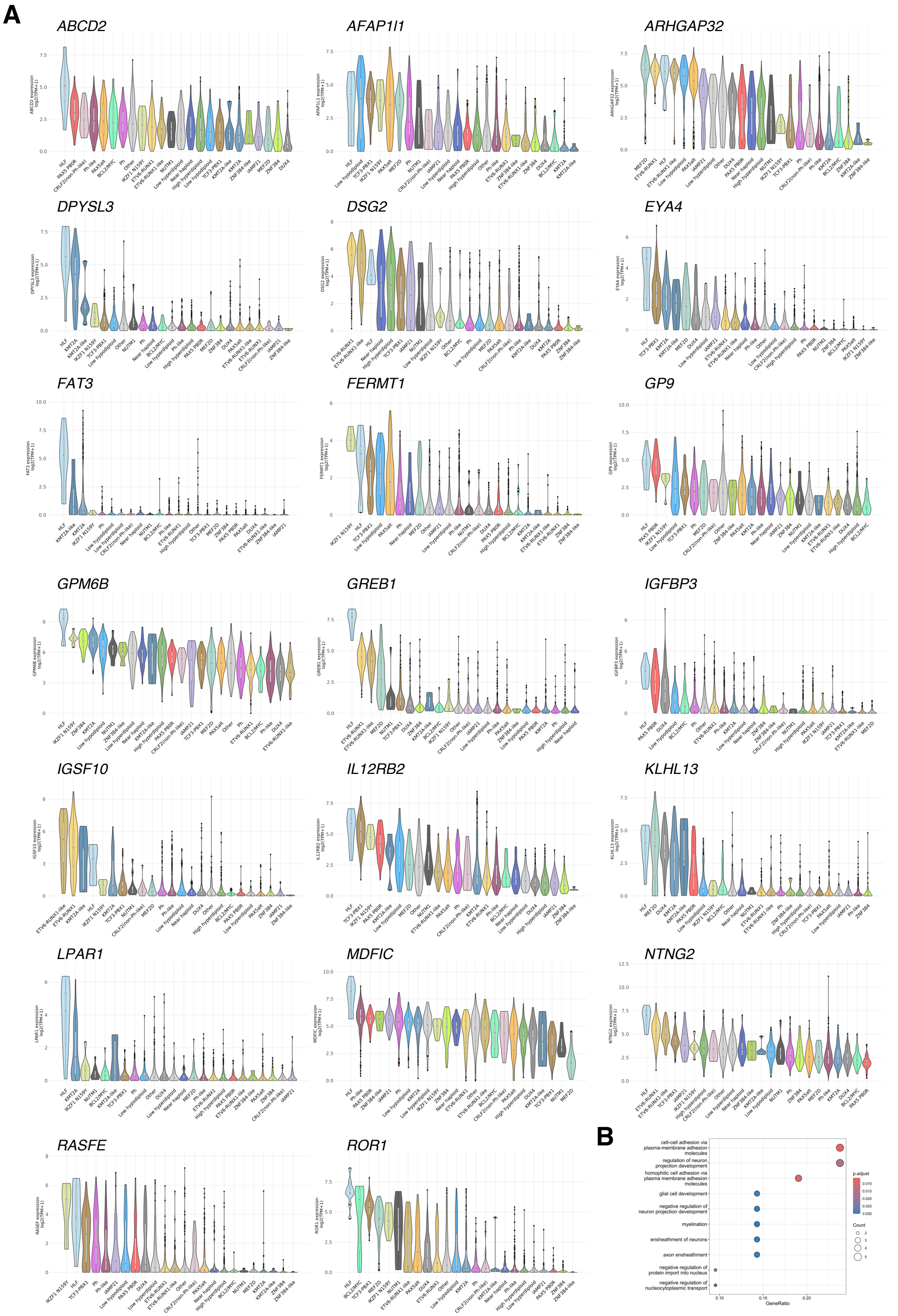
A
ABCD2
AFAP1L1
ARHGAP32
DPYSL3
DSG2
EYA4
FAT3
FERMT1
GP9
GPM6B
GREB1
IGFBP3
IGSF10
IL12RB2
KLHL13
LPAR1
MDFIC
NTNG2
RASFE
ROR1
B
cell-cell adhesion via plasma-membrane adhesion molecules
regulation of neuron projection development
homophilic cell adhesion via plasma membrane adhesion molecules
glial cell development
negative regulation of neuron projection development
myelination
ensheathment of neurons
axon ensheathment
negative regulation of protein import into nucleus
negative regulation of nucleocytoplasmic transport
GeneRatio
p.adjust
Count

Figure S2. E-P gene expression among ALL subtypes

(**A**) Violin plots showing gene expression levels of 22 E-P genes across different ALL subtypes showing the highest mean expression in the HLF cohort compared to other ALL subtypes. Data are from the St. Jude Cloud (https://www.stjude.cloud).

(**B**) Plots showing gene ontology terms for biological processes of E-P genes that are downregulated upon *TCF3::HLF*-KO and highest expression levels in TCF3::HLF (HLF) cohorts compared to other ALL subtypes. Details to GO analysis are found in **table S7**.

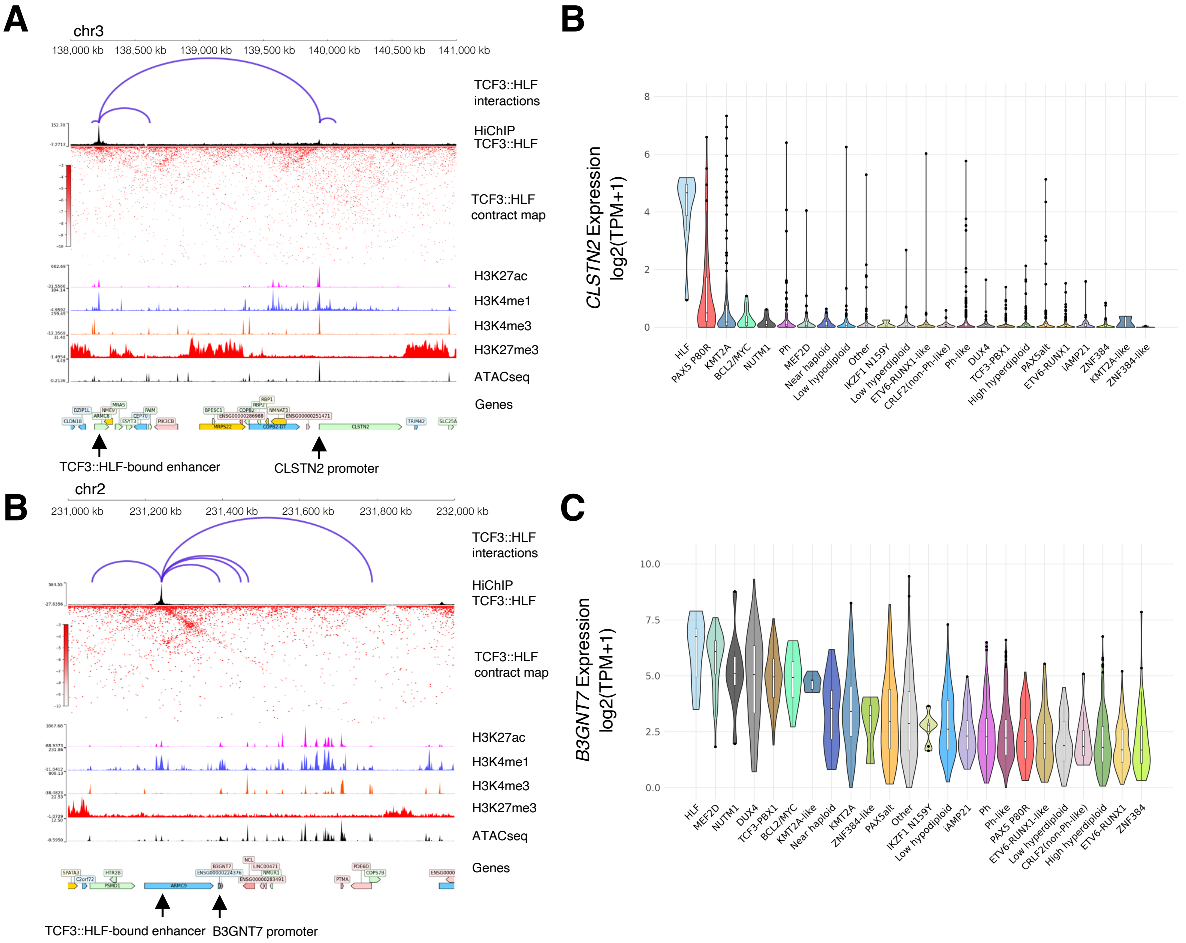

Figure S3. TCF3::HLF HiChIP pipeline

(**A,B**) Visualization of TCF3::HLF-associated interactions with *CLSTN2* (**A**) and *B3GNT7* (**B**) and the corresponding H3K27ac, H3K4me1, H3K4me3, and H3K27me3 ChIPseq and ATACseq profiles of HAL-01 cells. TCF3::HLF paired interactions identified by HiChIP are depicted as arcs. (**C,D**) Violin plots showing gene expression levels of *CLSTN2* (**C**) and *B3GNT7* (**D**) across different ALL subtypes. Data from the St. Jude Cloud (https://www.stjude.cloud) (*46*). HLF correspond to TCF3::HLF positive ALL

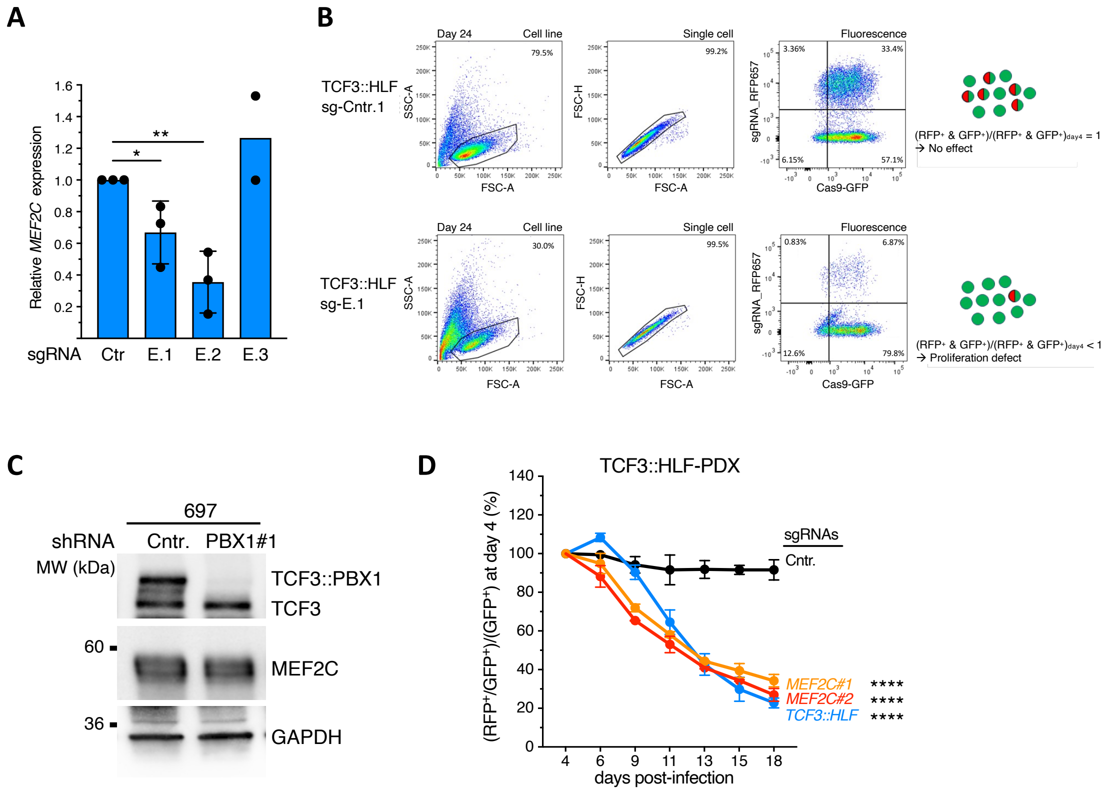

Figure S4. TCF3::HLF mediated regulation of MEF2C promotes expansion of leukemia cells
(**A**) RT-qPCR showing changes in *MEF2C* mRNA expression levels upon *MEF2C*-enhancer KO. Data were normalized to 18S and ACTB. Values are from three independent experiments. Statistical significance was calculated with unpaired T-test (* < 0.05; ** < 0.001).
(**B**) Scatterplot of competitive assay fluorescent output showing the comparative results at day 24 after transduction.
(**C**) Western blot showing that MEF2C levels upon TCF3::PBX1 depletion. Measurements were performed upon depletion of TCF3::PBX1 for 48 hours. GAPDH serves as a loading control.
(**D**) Competitive assay quantification in TCF3::HLF-PDX upon *MEF2C*-KO. Values correspond to the ratio between RFP^+/GFP^+ and GFP^+ cell number at the indicated days relative to the ratio between RFP^+/GFP^+ and GFP^+ cell number measured at day 4 post-transduction. Statistical significance (P-values) of two independent experiments was calculated with two-way repeated measures ANOVA (****< 0.0001).

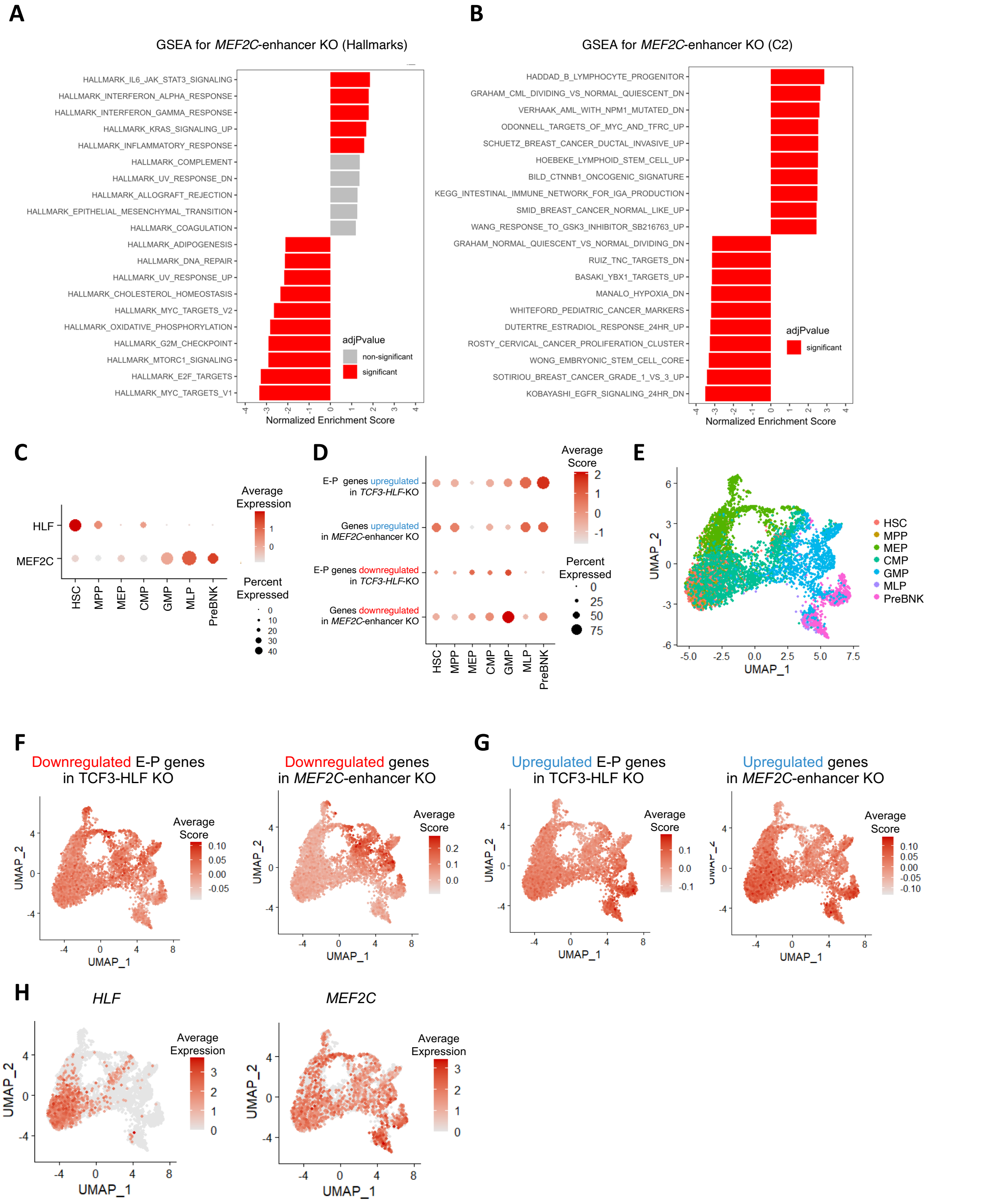

Figure S5. TCF3::HLF bound *MEF2C*-enhancer regulated gene signatures and expression of top genes in human bone marrow HSC and progenitors

(**A, B**) GSEA of upregulated and downregulated genes upon *MEF2C*-enhancer KO expression using HALLMARK and curated gene set from pathway databases and the biomedical literature

(C2.signature) (https://www.gsea-msigdb.org). The x-axis displays the normalized enrichment score with significant values (adj.p <0.05).

(**C**) Dot plot comparing the expression of *HLF* and *MEF2C* in the human bone marrow lymphoid, HSC and progenitor dataset by Pellin et al (*58*).

(**D**) Dot plot comparing module score for top 200 upregulated and downregulated genes (adj.p >0.05) upon *MEF2C*-enhancer KO or E-P genes up- and downregulated upon *TCF3::HLF*-KO in HAL-01 cells. Using data from Pellin et al. (58). HSC, hematopoietic stem cells; MPP, multipotent progenitors; MLP, multi-lymphoid progenitors; Pre-B/NK, Pre-B lymphocytes/natural killer cells; MEP, megakaryocyte-erythroid progenitors; CMP, common myeloid progenitors; GMP, granulocyte–monocyte progenitors). The color intensity and size of the dot plots correspond to average score and percent expression, respectively.

(**E**) UMAP analysis showing the clustering of scRNAseq of human bone marrow lymphoid, HSC and progenitor dataset.

(**F,G**) Human bone marrow lymphoid, HSC and progenitor scorecard dot plot showing top 200 upregulated (**F**) and downregulated genes (**G**) upon MEF2C-enhancer KO or from E-P genes differentially expressed upon *TCF3::HLF*-KO in HAL-01 cells.

(**H**) UMAP analysis highlighting MEF2C and HLF expression

Supplementary Tables

Table S1. FitHiChIP output for TCF3::HLF HiChIP data with expanded annotations.
FitHiChIP called interactions with annotations derived from Histone ChIPseq (normalized RPKM enrichment score), Deseq2 outputs for TCF3::HLF KO RNAseq (FC_Deseq, Log2 foldchange, adj.p), HAL-01 ATACseq (normalized RPKM enrichment score), Fantom5 database enhancer annotation, ROSE analysis annotation and Homer peakAnnotation. Histone landscape classification (cluster) is derived from deeptool ComputeMatrix feature hierarchical clustering described in Material & Methods. Annotations are done from left anchors (green columns) to right anchors (red columns) in genomic direction of arcs, FitHiChIP interaction annotation (blue columns).

Table S2. Summary of the stratum-adjusted correlation coefficient (SCC) between the TCF3 HiChIP replicates across all chromosomes.

Table S3. Motif analysis of TCF3::HLF interaction pairs not containing HLF motif.

Table S4. List of HiChIP identified E-P genes differentially expressed in TCF3::HLF KO and annotation of most significant interaction group.
Histone landscape classification (cluster) is derived from deeptool ComputeMatrix feature analysis for the TCF3::HLF-bound anchor site while the Pval_interaction, QValue_Bias, sumCC and InteractioID annotations are derived from the FitHiChIP output.

Table S5. Summary of identified E-P genes and associated interactions.

Table S6. List of TCF3::HLF KO differentially expressed TFs identified as E-P genes by HiChIP analysis followed by gene ontology analysis.

Table S7. Gene ontology analysis of E-P genes with high expression in TCF3::HLF positive leukemia presented in fig. S2.

Table S8. Differential expression of *MEF2C*-enhancer KO RNAseq, gene ontology terms of genes regulated upon MEF2C-enhancer KO and TCF3::HFL-KO, and GSEA using HALLMARK and c2.ALL signature.

Table S9. GSEA of *MEF2C*-enhancer KO and *TCF3::HLF*-KO expression profiles using custom signature.

Table S10. List of sgRNAs and primers.
